# Calidad de vida en mujeres con cáncer de mama sometidas a quimioterapia en Cali, Colombia

**DOI:** 10.7705/biomedica.4971

**Published:** 2020-06-30

**Authors:** María Elena Mejía-Rojas, Adolfo Contreras-Rengifo, Mauricio Hernández-Carrillo

**Affiliations:** 1 Escuela de Enfermería, Grupo de Investigación en Cuidado de Enfermería, Universidad del Valle, Cali, Colombia Escuela de Enfermería, Grupo de Investigación en Cuidado de Enfermería Universidad del Valle, Cali Colombia; 2 Escuela de Odontología, Grupo de Investigación en Medicina Periodontal, Universidad del Valle, Cali, Colombia Escuela de Odontología, Grupo de Investigación en Medicina Periodontal Universidad del Valle Cali Colombia; 3 Doctorado en Salud, Universidad del Valle, Cali, Colombia Doctorado en Salud Universidad del Valle Cali Colombia

**Keywords:** neoplasias de mama, calidad de vida, quimioterapia, salud de la mujer, Colombia, Breast neoplasms, quality of life, drug therapy, women’s health, Colombia

## Abstract

**Introducción.:**

El cáncer de mama es una neoplasia grave que se origina en los tejidos mamarios y cuyo tratamiento demanda quimioterapia, con los consecuentes cambios en la calidad de vida.

**Objetivo.:**

Determinar los factores de riesgo asociados con la calidad de vida relacionada con la salud en mujeres con cáncer de mama sometidas a quimioterapia en Cali, Colombia.

**Materiales y métodos.:**

Se hizo un estudio observacional y transversal con componente analítico en una muestra de 80 mujeres, utilizando los cuestionarios QLQ-C30 para cáncer y QLQ-BR23 para cáncer de mama, así como información sociodemográfica y clínica. Se hizo un análisis de regresión logística para determinar los factores asociados con las razones de momios (*odd ratios*, OR) ajustadas y un intervalo de confianza (IC) de 95 %; la calidad de vida se clasificó mediante el cuestionario QLQ-BR23.

**Resultados.:**

Los síntomas más relevantes fueron fatiga, insomnio y pérdida de cabello. Se redujeron la funcionalidad física, las sensaciones de placer y la actividad sexual. Los factores asociados con la baja calidad de vida fueron los síntomas mamarios (OR ajustada=5,5; IC_95%_ 1,2-24,8; p=0,038), los efectos secundarios del tratamiento sistémico (OR ajustada=7,3; IC_95%_ 2,6-22,1; p=0,012), un menor placer sexual (OR ajustada=1,8; IC_95%_ 1,2-11,8; p=0,027) y la reducción de expectativas para el futuro (OR ajustada=4,2; IC95% 1,1-17,8; p=0,045).

**Conclusiones.:**

En las mujeres con cáncer de mama sometidas a quimioterapia, la calidad de vida se vio afectada principalmente por los efectos secundarios del tratamiento, en tanto que los signos y los síntomas más relevantes fueron la pérdida del cabello, el insomnio y la fatiga, además de los síntomas mamarios, la menor funcionalidad física y la menor sensación de placer sexual. Se recomienda la implementación de estrategias de intervención destinadas a mejorar la calidad de vida, y el cuidado físico y emocional de las pacientes.

## Abstract (en)

**Introduction:**

Breast cancer is a serious neoplasm that originates in mammary tissues whose treatment with chemotherapy seems to generate significant changes in the quality of life.

**Objective:**

To determine the risk factors associated with health-related quality of life in women with breast cancer under chemotherapy in Cali, Colombia.

**Materials and methods:**

We conducted an observational, cross-sectional study with analytical components in a sample of 80 female patients using the QLQ-C30 questionnaire for cancer, and the QLQ-BR23 for breast cancer, as well as sociodemographic and clinical information. We performed a logistic regression analysis to determine associated risk factors through adjusted OR and 95% CI; the quality of life was classified using the QLQ-BR23.

**Results:**

The most relevant symptoms were fatigue, insomnia, and hair loss. Physical functionality, feelings of pleasure, and sexual activity were reduced. The factors associated with low quality of life were mammary symptoms (adjusted OR= 5.5; 95% CI: 1.2-24.8; p = 0.038), systemic therapy side effects (adjusted OR = 7.3; IC95%: 2.6-22.1; p = 0.012), lower sexual pleasure (adjusted OR = 1.8; IC95%: 1.2-11.8; p = 0.027) and reduction of expectations about the future (adjusted OR = 4.2; IC95%: 1.1-17.8; p = 0.045).

**Conclusions:**

The quality of life in women with breast cancer and under chemotherapy was mainly affected by the side effects of the therapy; the most relevant signs and symptoms identified were hair loss, insomnia, and fatigue besides breast symptoms, lower physical functionality, and decreased sensation of sexual pleasure. We recommend intervention strategies aimed at improving the quality of life and the physical and emotional care of these patients.

El cáncer de mama es una neoplasia maligna que requiere tratamiento radical y un diagnóstico temprano; el tumor se extirpa si está localizado, pues produce metástasis y, eventualmente, la muerte. Afecta principalmente a las mujeres y, muy ocasionalmente, a los hombres ^1^. Según el informe mundial de cáncer del *Global Cancer Observatory* en el 2018, la Organización Mundial de la Salud (OMS) reporta el cáncer como la principal causa de muerte a escala mundial. Se estima que cerca de 18 millones de casos nuevos se diagnostican cada año en el mundo ^2^. Según cifras del Instituto Nacional de Cancerología, en el 2018 se presentaron 101.893 casos nuevos de cáncer en Colombia, 46.057 muertes por todos los tipos de tumores y el número de casos prevalentes a cinco años fue de 230.726 enfermos ^3^. Según el Registro Poblacional de Cáncer de Cali, en el 2019 se reportaron 754 casos nuevos de cáncer de mama en Cali, es decir, el 23,3 % de todos los tipos de cáncer ^4^.

La mayoría de las muertes se produce en los países de ingresos bajos y medios, en donde las mujeres con cáncer de mama son diagnosticadas en estadios avanzados debido a la falta de sensibilización sobre la detección precoz y a los obstáculos en el acceso a los servicios de salud ^5^. En Latinoamérica y el Caribe, el cáncer de mama es el más común entre las mujeres y el segundo en mortalidad, con el mayor porcentaje de muertes en mujeres menores de 65 años (56 %), en comparación con los Estados Unidos y Canadá (37 %) ^6^.

El cáncer de mama en Colombia ha ido en aumento en los últimos cinco años. Es la tercera causa de muerte por cáncer en mujeres, después del cáncer de cuello uterino y de estómago ^7^. Según el “Atlas de mortalidad por cáncer”, en Colombia se diagnostican al año 5.526 casos y ocurren 2.253 fallecimientos por esta enfermedad, lo que equivale a 15 nuevos diagnósticos y seis muertes por día ^8^.

Hay grandes diferencias en las tasas de la enfermedad debido a la variación en los factores de riesgo, el tipo de comportamiento reproductivo, el nivel socioeconómico y los antecedentes familiares ^9^. En el 2019, se esperaba que los servicios oncológicos de Cali diagnosticaran aproximadamente 9.800 nuevos casos de cáncer ^10^.

Aunque algunas mujeres enfrentan positivamente la experiencia del tratamiento, otras pueden sentirse emocionalmente afectadas debido a cambios en su imagen corporal y en su feminidad. Debido a la mortalidad relativamente alta y a la necesidad de un tratamiento agresivo de un año duración, el diagnóstico de cáncer de mama tiene un gran impacto en la vida de las sobrevivientes. El apoyo familiar y el de la pareja parecen minimizar los efectos secundarios de la quimioterapia, los cuales pueden ocasionar cambios significativos en la calidad de vida. Sin embargo, este importante apoyo con frecuencia desaparece a lo largo del tiempo ^11^, por lo que debe recalcarse su importancia para las pacientes con cáncer.

En este contexto, el presente estudio se propuso determinar la calidad de vida en el marco de su relación con la salud en mujeres con cáncer de mama sometidas a la primera quimioterapia.

Las personas enfrentan diversos cambios durante el proceso de enfermedad, entre ellos el aislamiento social que incrementa los riesgos de las mujeres con cáncer de mama. En este sentido, un estudio previo evidenció que un ambiente social negativo produce un mayor crecimiento tumoral ^12^.

El diagnóstico de cáncer y la quimioterapia enfrentan a las pacientes con nuevos roles sociales y la necesidad de sobreponerse a sus efectos deletéreos ^13^. Con frecuencia se produce un cierto aislamiento social acompañado de sentimientos de tristeza. Por ejemplo, la quimioterapia incluye doxorrubicina, un medicamento con muchos efectos secundarios, entre ellos, la alopecia ^14^. lo que exige el uso de pelucas o de gorros, prendas que ya hacen parte del estereotipo social de “paciente con cáncer”. Al sentir más cercana la muerte, las mujeres con cáncer de mama preparan a su familia y tratan de minimizar los efectos de la enfermedad y del tratamiento ^15^.

En un estudio en el que se valoraron las experiencias de vida de mujeres con cáncer de mama sometidas a quimioterapia, se encontró que el aislamiento social comienza con el tratamiento, pues dado que la quimioterapia afecta el sistema inmunitario, los médicos tratantes recomiendan reducir las visitas y las salidas para evitar el riesgo de infección ^16^. También, la deshidratación y los efectos citotóxicos sobre órganos y tejidos contribuyen a la pérdida de cabello y de la tersura de la piel, factores que afectan la feminidad y la autoestima de la mujer, por lo que se recomienda la intervención psicológica antes y después de la quimioterapia ^17^. El apoyo familiar y la relación con la pareja son puntos claves en el tratamiento y la recuperación de las mujeres, y se ha reportado su asociación con la supervivencia y un mejor desempeño sexual ^18^.

La calidad de vida basada en el bienestar, la felicidad y la satisfacción de un individuo, incentiva su capacidad de actuación y le ofrece una sensación positiva de la vida. Se trata de un concepto amplio y muy subjetivo que se ve influenciado por la salud física y mental, el grado de dependencia física y la fortaleza de los círculos sociales de apoyo ^19^. A pesar de los efectos negativos en la calidad de vida de los pacientes con cáncer, la quimioterapia elimina las células cancerosas y reduce el riesgo de metástasis ^20^.

Este estudio se guió por la teoría de Peterson y Bredow sobre la calidad de vida relacionada con la salud, quienes la conciben como separada de la calidad global de vida ^19^. La calidad de vida es multidimensional, multifacética y es útil en la clínica. El modelo de Peterson y Bredow permite valorar la calidad de vida relacionada con la salud según la realidad social y cultural de Colombia, por medio de los cuestionarios QLQ-C30 y QLQ-BR23, aplicados en varios estudios como el de Arraras-Urdaniz, *et al*. ^21^.

En este contexto, el propósito del presente estudio fue determinar los factores de riesgo asociados con la calidad de vida relacionada con la salud en mujeres con cáncer de mama sometidas a quimioterapia en Cali, Colombia. Los resultados aspiran a contribuir al mejoramiento de las políticas de salud orientadas a la solución de los problemas de las mujeres con cáncer de mama que reciben quimioterapia.

## Materiales y métodos

Se hizo un estudio observacional y transversal con componente analítico de asociación de 80 mujeres adultas con cáncer de mama que recibían quimioterapia en un hospital público y en dos instituciones privadas de Cali.

Los criterios de inclusión fueron el ser mujer con diagnóstico de cáncer de mama y estar recibiendo quimioterapia. Los criterios de exclusión fueron el tratamiento con radioterapia o la mastectomía. Se hizo un muestreo no probabilístico a partir de las bases de datos institucionales y se invitó a participar en la investigación a 53 pacientes del Hospital Universitario del Valle, a 18 del Centro Médico Imbanaco y a 9 de Funcancer.

Se recolectaron los datos a partir del segundo semestre del 2017 y hasta junio de 2018, mediante un cuestionario sociodemográfico y dos cuestionarios de calidad de vida de la *European Organisation for Research and Treatment of Cáncer* (EORTC) para pacientes con cáncer: la QLQ-C30 y la QLQ-BR23 (específica para cáncer de mama), ambas con pruebas psicométricas validadas en Colombia ^20^.

El cuestionario QLQ-C30 para pacientes con cáncer está compuesto por 30 ítems divididos en cinco dimensiones funcionales: física, cognitiva, emocional, de desempeño social, y de capacidad para realizar tareas diarias relacionadas con el papel funcional; en tres demisiones de síntomas: fatiga, náuseas y dolor; en seis elementos individuales: disnea, insomnio, pérdida de apetito, estreñimiento y diarrea, y en dos preguntas sobre la salud en general.

El QLQ-BR 23 es específico para pacientes con cáncer de mama y se compone de 23 ítems incorporados en la medición de los efectos secundarios de las diferentes modalidades de tratamiento; incluye, además, aspectos relacionados con la quimioterapia y los síntomas mamarios, la imagen corporal, la función sexual y las perspectivas futuras. También, se utilizó un cuestionario en el que se indagaba sobre la información socioeconómica, la situación laboral, los antecedentes clínicos, los hábitos y el estilo de vida.

Como variable dependiente, se usó la valoración de la calidad de vida a partir del resultado del cuestionario QLQ-BR23, teniendo en cuenta el puntaje global obtenido ^21^, el cual se dividió con base en la suma del puntaje de los 23 ítems en la escala de Likert y la estandarización en un puntaje que varió de 0 a 100, siendo el puntaje alto el que indicaba una mejor percepción de la calidad de vida. Las pacientes con valores inferiores o iguales a 50 correspondían a aquellas con mala percepción de la calidad de vida (valor 1), en tanto que los puntajes por encima de 50 correspondieron a pacientes que percibían su calidad de vida como buena (valor 0) ^22^. En la muestra de 80 pacientes, 40 tenían una percepción negativa de su calidad de vida, y las otras 40 percibían su calidad de vida como buena.

Las variables explicativas para la determinación de los factores de riesgo incluían las sociodemográficas y los dominios de los cuestionarios QLQ-C30 y QLQ-BR23 descritos como sociodemográficos: edad, número de hijos, estado marital, estrato socioeconómico, escolaridad, escolaridad completa, situación laboral, y ocupación. Las variables clínicas y las relacionadas con los servicios de salud, incluyeron: institución, afiliación al sistema de salud, tiempo del diagnóstico (meses), recurso de la tutela, antecedentes familiares de cáncer de mama, progresión de la enfermedad, cirugía, estadio del tumor, clasificación histopatológica, receptores de estrógenos y progesterona, uso de tamoxifeno, dosis formulada si se recibía algún tipo de medicamento, y los marcadores tumorales Her2, Brca y Fish.

Con el cuestionario QLQ-C30, se midieron las variables funcionales físicas, cognitivas, emocionales y de desempeño social, y la capacidad para realizar tareas diarias, así como tres demisiones de síntomas: fatiga, náuseas y dolor; seis elementos individuales: disnea, insomnio, pérdida de apetito, estreñimiento, diarrea y dificultades financieras, y dos preguntas sobre la salud en general.

Los dominios del cuestionario QLQ-BR23 fueron: imagen corporal, funcionamiento sexual, placer sexual, perspectivas de futuro, efectos secundarios de la terapia sistémica, síntomas mamarios, síntomas braquiales y pérdida de cabello.

El análisis estadístico se hizo con el programa Stata^™^, versión 14, y en cuadros y gráficos de Excel.

### Consideraciones éticas

El estudio fue avalado por el Comité Institucional de Revisión de Ética Humana de la Universidad del Valle según acta de aprobación 012014. En cuanto al uso de los cuestionarios, se obtuvo la firma del consentimiento informado de todas las participantes.

## Resultados

Al evaluar las variables de edad (p=0,8232) y de número de hijos (p=0,0116), solo en la primera se obtuvo normalidad. La edad promedio de las pacientes fue de 52,7 ± 12,1 años en aquellas con percepción negativa de su calidad de vida y de 54,9 ± 12,4 en aquellas con una positiva; los promedios de las escalas funcionales del QLQ C30 y el QLQ BR23 fueron 65,4 ± 15,6 y 46,9 ± 12,5, respectivamente.

En cuanto a las variables sociodemográficas, se determinaron las frecuencias relativas de cada categoría con los siguientes resultados: 37,5 % correspondía a mujeres casadas, 28,8 % había cursado la primaria, 36,3 % había completado la secundaria, y 60 % de las pacientes estaba trabajando.

Con respecto al estrato socioeconómico, los más prevalentes fueron el 2 y 3, con 46,3 % y 26,3 %, respectivamente. El 70 % de las pacientes había completado su escolaridad; la mediana del número de hijos fue 2 (0 a 4) y, en cuanto al estado marital, no se evidenciaron diferencias estadísticamente significativas entre las distintas categorías (p=0,671), como tampoco en el estrato socioeconómico (p=0,598) ni en la distribución según escolaridad, con un valor de probabilidad cercano a 1, lo que indica que el comportamiento de esta variable fue significativamente igual independientemente de la percepción que las pacientes tenían de su calidad de vida.

En cuanto a si tenían escolaridad completa, se encontró un resultado similar en ambos grupos (p=0,626). La situación laboral también era similar en ambos grupos de pacientes, pues la mayoría se encontraba activa y en la comparación no se obtuvieron diferencias estadísticamente significativas (p=0,168). La ocupación más frecuente fue la de ama de casa, con una frecuencia de 19 pacientes en cada uno de los grupos, y sin diferencias estadísticamente significativas (p=0,541) (cuadro 1).

**Cuadro 1:** Descripción y comparación de las variables sociodemográficas según la percepción de la calidad de vida con el cuestionario QLQ-BR23 en mujeres sobrevivientes de cáncer de mama sometidas a quimioterapia, Cali, 2017

Variable/Categoría*		Mala calidad de vida (n=40)		Buena calidad de vida (n=40)		p	Total	
		n	%	n	%		n	%
Estado marital	Casada	15	18,8	15	18,8	0,671	30	37,5
	Otro	4	5	5	6,3		9	11,3
	Soltera	10	12,5	8	10		18	22,5
	Unión libre	10	12,5	8	10		18	22,5
	Viuda	1	1,3	4	5		5	6,3
Estrato	1	4	5,0	4	5,0	0,598	8	10,0
socioeconómico	2	20	25,0	17	21,3		37	46,3
	3	12	15,0	9	11,3		21	26,3
	4	3	3,8	6	7,5		9	11,3
	5	1	1,3	3	3,8		4	5,0
	6	0	0,0	1	1,3		1	1,3
Escolaridad	Primaria	11	13,8	12	15,0	0,956	23	28,8
	Bachillerato	16	20,0	13	16,3		29	36,3
	Técnico	2	2,5	3	3,8		5	6,3
	Universidad	7	8,8	7	8,8		14	17,5
	Posgrado	4	5,0	5	6,3		9	11,3
Escolaridad	No	11	13,8	13	16,3	0,626	24	30,0
completa	Sí	29	36,3	27	33,8		56	70,0
Situación laboral	Activa	28	35,0	20	25,0	0,168	48	60,0
	Incapacitada	5	6,3	4	5,0		9	11,3
	Jubilada	1	1,3	1	1,3		2	2,5
	Otros	6	7,5	12	15,0		18	22,5
	Pensionada	0	0,0	3	3,8		3	3,8
Ocupación	Ama de casa	19	23,8	19	23,8	0,541	38	47,5
	No registra	0	0	2	2,5		2	2,5
	Trabajo formal	14	17,5	12	15		26	32,5
	Trabajo informal	7	8,8	7	8,8		14	17,5
**Cuantitativas**		**Mala calidad de vida**		**Buena calidad de vida**			**p**	
Edad (años)^μ^		52,7 ±12,1		54,9 ± 12,4			0,43	
Número de hijos^†^		2 (0 - 4)		2 (0 - 4)			0,675	

* Comparación de proporciones en la prueba de ji al cuadrado

^µ^ Media ± DE en la prueba t de Student de muestras independientes

^†^ Mediana (RIC) en la prueba U de Mann-Whitney

En cuanto a las variables clínicas, el estadio del tumor era avanzado en 3,8 % de las pacientes y era incipiente en 66,2 %. Entre las pacientes con el tumor en un estadio avanzado, el 25 % correspondía a la categoría IIB. Con relación al tipo de quimioterapia suministrada a las pacientes, la combinación de adriamicina y ciclofosfamida fue la más frecuente (22,5 %). La clasificación histopatológica más común fue la de carcinoma ductal infiltrativo, con 83,8 %. Los receptores de estrógenos y progesterona presentaron igual porcentaje de positivos y negativos (50 %). En cuanto a los marcadores tumorales, 56,2 % fue negativo para Her2, 96,2 % lo fue para el Brca, y 86,2 % lo fue para el índice Fish. El 8,8 % de las pacientes se clasificó clínicamente en el momento del diagnóstico. El valor más prevalente del índice de Karnofsky fue 90 %, con una frecuencia relativa de 76,3 %. Del 28,7 % que recibió tamoxifeno, un alto porcentaje tenía una percepción positiva de su calidad de vida (20 %) en comparación con aquellas cuya percepción era negativa (8,8 %). Además, en cuanto al tipo de quimioterapia administrada, se observaron diferencias estadísticamente significativas entre los grupos (p<0,0001) (cuadro 2).

**Cuadro 2:** Descripción y comparación de las variables clínicas según la percepción de la calidad de vida con el cuestionario QLQ-BR23 en mujeres sobrevivientes de cáncer de mama sometidas a quimioterapia en Cali, 2017

Variable	Categoría*	Mala calidad de vida (n=40)		Buena calidad de vida (n=40)		p	Total	
		n	%	n	%		n	%
Estadio del tumor	Avanzado	16	20,0	11	13,8	0,237	27	33,8
	Inicial	24	30,0	29	36,2		53	66,2
Primer estadio	l	4	5,0	3	3,8	0,328	7	8,8
	llA	6	7,5	13	16,3		19	23,8
	llB	11	13,8	9	11,3		20	25,0
	lllA	7	8,8	7	8,8		14	17,5
	lllB	6	7,5	3	3,8		9	11,3
	lllC	0	0,0	2	2,5		2	2,5
	lV	6	7,5	3	3,8		9	11,3
Tipo de	Adriamicina	5	6,3	13	16,3	< 0,0001	18	22,5
quimioterapia	Doxorrubicina	0	0,0	16	20,0		16	20,0
administrada	Taxol	9	11,3	4	5,0		13	16,3
	AC-TH	9	11,3	3	3,8		12	15,0
	Trastuzumab	9	11,3	1	1,3		10	12,5
	Paclitaxel	5	6,3	2	2,5		7	8,8
	Adyuvante TC	1	1,3	1	1,3		2	2,5
	Anastrazol	1	1,3	0	0,0		1	1,3
	FAC-Trastaza	1	1,3	0	0,0		1	1,3
Clasificación	Adenocarcinoma	1	1,3	0	0,0	0,615	1	1,3
histopatológica	Carcinoma ductal	32	40,0	34	42,5		66	82,5
	Carcinoma lobular	7	8,8	6	7,5		13	16,3
Receptores de	No	19	23,8	21	26,3	0,655	40	50,0
estrógeno y	Sí	21	26,3	19	23,8		40	50,0
progesterona								
Marcador tumoral	Negativo	20	25,0	25	31,3	0,260	45	56,3
Her2	Positivo	20	25,0	15	18,8		35	43,8
Marcador	Negativo	37	46,3	40	50,0	0,077	77	96,3
tumoralBrca	Positivo	3	3,8	0	0,0		3	3,8
Marcador tumoral	Negativo	36	45,0	33	41,3	0,330	69	86,3
Fish	Positivo	4	5,0	7	8,8		11	13,8
Recibe tamoxifeno	No	33	41,3	24	30,0	0,026	57	71,3
	Sí	7	8,8	16	20,0		23	28,8
Clasificación	l	4	5,0	3	3,8	0,590	7	8,8
clínica en el	ll	16	20,0	19	23,8		35	43,8
momento del	lll	15	18,8	14	17,5		29	36,3
diagnóstico	lV	5	6,3	2	2,5		7	8,8
	A	0	0,0	1	1,3		1	1,3
	B	0	0,0	1	1,3		1	1,3
Índice de	70	0	0,0	1	1,3	0,145	1	1,3
Karnofsky (%)	80	2	2,5	1	1,3		1	1,3
	90	27	33,8	34	42,5		3	3,8
	100	11	13,8	4	5,0		61	76,3
							15	18,8

* Comparación de proporciones en la prueba de ji al cuadrado

Con relación a las variables relacionadas con la atención en salud, hubo diferencias estadísticamente significativas entre las pacientes con una percepción negativa de su calidad de vida y el grupo de quienes tenían una percepción positiva, dependiendo del tipo de institución (pública o privada) donde eran atendidas (p=0,002). Asimismo, el 67,5 % de las pacientes mencionaron haber pasado por cirugía, variable que tuvo una relación estadística con diferencias significativas (p=0,004) entre los grupos de análisis. En cuanto a la afiliación al sistema de salud, el 63,8 % de las pacientes pertenecía al régimen contributivo y el 36,2 % al subsidiado (p=0,816). Con relación a la acción de tutela, el 13,8 % había acudido a este mecanismo (p=0,330). No había evidencia de antecedentes familiares en el 68,7 % (p=0,469), en tanto que el 31,3 % tenía dichos antecedentes. La progresión de la enfermedad se reportó en 13,8 % de las pacientes del estudio (p=0,105). El promedio en meses para el diagnóstico fue de 11,9 ± 6,7 y 18,7 ± 28,21 en las pacientes con mala y buena calidad de vida, respectivamente, lo que indica una gran variabilidad en dicho tiempo, sin evidenciarse diferencias significativas entre los grupos (p=0,1424). En cuanto a la dosis formulada en miligramos, se obtuvo un promedio de 7,1 ± 9,6 en las pacientes con mala calidad de vida y de 1,1 ± 4,4 en aquellas con buena calidad de vida (p=0,0007) (cuadro 3).

**Cuadro 3:** Descripción y comparación de las variables relacionadas con la atención en salud según la percepción de la calidad de vida en el cuestionario QLQ-BR23 en mujeres sobrevivientes de cáncer de mama sometidas a quimioterapia en Cali, 2017

Variable	Categoría	Mala calidad de vida (n=40)		Buena calidad de vida (n=40)		p	Total	
		n	%	n	%		n	%
Institución	Privada	7	8,8	20	25,0	0,002	27	33,8
	Pública	33	41,3	20	25,0		53	66,2
Afiliación a salud	Contributivo	25	31,3	26	32,5	0,816	51	63,8
	Subsidiado	15	18,8	14	17,5		29	36,2
Tutela	No	36	45,0	33	41,3	0,33	69	86,2
	Sí	4	5,0	7	8,8		11	13,8
Antecedentes familiares	No	29	36,3	26	32,5	0,469	55	68,8
de cáncer de mama	Sí	11	13,8	14	17,5		25	31,2
Progresión de la	No	37	46,3	32	40,0	0,105	69	86,2
enfermedad	Sí	3	3,8	8	10,0		11	13,8
Cirugía	No	7	8,8	19	23,8	0,004	26	32,5
	Sí	33	41,3	21	26,3		54	67,5
**Cuantitativas**			**Mala calidad de vida**	**Buena calidad de vida**			**p**	
Tiempo del diagnóstico (meses)μ			11,9 ± 6,7	18,7 ± 28,2			0,1424	
Dosis formulada (mg)μ			7,1 ± 9,6	1,1 ± 4,4			0,0007	

* Comparación de proporciones en la prueba de ji al cuadrado

µ Media ± DE en la prueba de t de Student de muestras independientes

**Figura 1:**
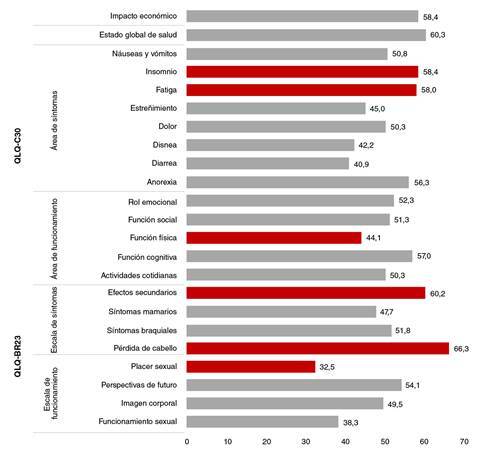
Distribución de los puntajes según el cuestionario y la dimensión en mujeres sobrevivientes de cáncer de mama sometidas a quimioterapia en Cali, 2017

En el QLQ-C30 se obtuvieron puntuaciones menores que en el QLQ- BR23 en cuanto a la presencia de síntomas. En general, las mujeres registraron como buena la valoración del estado global de salud (58,4) y la del impacto económico (60,3), lo que demostró su capacidad para continuar desempeñando las actividades en el hogar y en su trabajo. En lo relacionado con los síntomas, en el cuestionario QLQ-C30, se observó que el insomnio y la fatiga fueron los de mayor frecuencia, con puntajes de 58,4 y 58,0, respectivamente. En el área de funcionamiento, se obtuvo un puntaje de 44,1 para la función física. En el QLQ-BR23, el área de síntomas fue la más afectada: los efectos secundarios obtuvieron un puntaje de 60,2 y la pérdida de cabello, uno de 66,3. Con respecto a la escala de funcionamiento, el placer sexual fue el más afectado, con un valor de 32,5, siendo este un aspecto muy relevante para las participantes en el estudio (figura 1).

En el análisis multivariado se tuvieron en cuenta varios modelos y se incluyeron variables sociodemográficas, clínicas y las relacionadas con la atención en salud. Se establecieron como candidatas aquellas cuyos valores de probabilidad estuvieron por debajo de 0,25, de tal manera que estas se conservaron hasta culminar el proceso de modelación. Se encontró como modelo final para la calidad de vida relacionada con la salud en los pacientes con cáncer de mama que las dimensiones que más inciden en dicho deterioro son los efectos secundarios de la quimioterapia, la función sexual, los síntomas mamarios y la deficiente perspectiva de futuro después del ajuste (cuadro 4).

**Cuadro 4:** Modelo explicativo de los factores asociados con la calidad de vida en pacientes sobrevivientes de cáncer de mama sometidas a quimioterapia en Cali, 2017

Variable	OR cruda (IC_95%_)	OR ajustada (IC_95%_)
Estado laboral	0,82 (0,63 - 1,06)	1,60 (0,53 - 4,75)
Estrato socioeconómico	0,74 (0,48 - 1,14)	7,20 (0,22 - 19,02)
Cirugía	4,27 (1,53 - 1,89)*	1,10 (0,70 - 8,62)
Recibe tamoxifeno	3,14 (1,12 - 8,82)*	0,30 (0,04 - 1,6)
Imagen corporal	2,40 (1,67 - 3,44)*	1,40 (0,84 - 11,07)
Función sexual	1,16 (0,77 - 1,75)	1,80 (1,23 - 11,83)*
Perspectivas para el futuro	3,63 (1,99 - 6,58)*	4,20 (1,13 - 17,78)*
Síntomas mamarios	1,92 (1,45 - 2,54)*	5,50 (1,24 - 24,81)*
Síntomas braquiales	2,50 (1,69 - 3,69)*	12,30 (0,53 - 28,5)
Pérdida del cabello	3,82 (2,12 - 6,86)*	0,40 (0,00 - 15,91)
Efectos secundarios	1,89 (1,42 - 2,51)*	7,30 (2,55 - 22,07)*

OR: *odds ratio*

* Significativos: 0,05;

Después de ajustar a partir de los modelos de regresión logística, los aspectos que más afectaron la calidad de vida de las pacientes fueron la función sexual (OR ajustada=1,8; IC_95%_ 1,2-11,8; p=0,027), las perspectivas para el futuro (OR ajustada=4,2; IC_95%_ 1,1-17,8; p=0,045), los síntomas mamarios (OR ajustada=5,5; IC_95%_ 1,2-24,8; p=0,038), y los efectos secundarios (OR ajustada=7,3; IC_95%_ 2,6-22,1; p=0,012). Las intervenciones, por lo tanto, deben centrarse en estos factores.

## Discusión

Las mujeres participantes se vieron afectadas en las áreas de funcionamiento y de los síntomas. En cuanto a la dimensión relativa a la sexualidad en la percepción de la calidad de vida, se encontró que el 52,5 % tuvo una percepción negativa (p=0,032). Según el estudio de Coady, *et al.*^23^, para el manejo de los problemas sexuales en pacientes con cáncer de mama debe comprenderse la fisiopatología del dolor sexual, el cual puede estar asociado con el tratamiento brindado a las pacientes. Estos tratamientos oncológicos pueden generar problemas en la respuesta sexual al afectar las emociones, el deseo sexual y los componentes centrales o periféricos del sistema nervioso, vascular pélvico y el eje hipotálamo-hipófisis- gónadas ^(23)^, aunque son más frecuentes cuando hay un conflicto marital. El diagnóstico del cáncer no produce trastornos en parejas felices, pero puede ocasionar conflictos y resultar en relaciones disfuncionales. Es de gran relevancia apreciar la vida en pareja y el apoyo de la familia de las mujeres con cáncer sometidas a quimioterapia ^12^, pues la sexualidad y la relación de pareja tienen un papel muy importante en el proceso de adaptación a la quimioterapia, puesto que esta altera la esfera cognitivo-afectiva y reduce la fortaleza física y la mental.

En relación con las perspectivas para el futuro, convivir con el cáncer de mama genera un estado de inseguridad que requiere de una actitud positiva para adaptarse y sobrevivir; dicha actitud pesa especialmente en la toma de decisiones, la predicción sobre el avance en su tratamiento, la tolerancia, el control, el estrés, la adaptación en su nueva forma de ver y vivir la vida, y la incertidumbre que genera sobrevivir con una enfermedad crónica ^23^. En este sentido, es pertinente profundizar en esta área tan poco explorada desde la perspectiva del proceso de salud y enfermedad ^25^ teniendo en cuenta que, frente a una enfermedad tan grave como el cáncer, algunas personas la asumen como una oportunidad e, incluso, desarrollan una nueva y positiva perspectiva de vida. Hubo correlación entre los síntomas y la percepción de la calidad de vida (p=0,012). En otro estudio, al evaluar cómo afectaba el tratamiento la calidad de vida de mujeres con diagnóstico de cáncer de mama, se encontró que el tratamiento mejoraba el funcionamiento físico ^26^.

En un estudio en Bogotá, los trastornos digestivos y alimentarios, la debilidad, el malestar general y las alteraciones en la integridad de la piel, fueron los síntomas más relacionados con la quimioterapia ^27^, lo que concuerda con los resultados del presente estudio, en el cual el vómito, el estreñimiento y el cansancio fueron los síntomas que más afectaron la calidad de vida de las pacientes. En diversos estudios se recomienda la interrupción del tratamiento como una buena práctica clínica cuando la paciente está en estado terminal ^28^, y en otros casos se sugieren intervenciones que minimicen los efectos adversos durante la quimioterapia ^29^^,^^30^, ya que, aunque su propósito es eliminar o reducir el volumen del tumor, el tratamiento con citotóxicos deteriora la salud de la paciente y causa náuseas, vómito, cansancio, anemia, pérdida temporal de cabello (alopecia) y un profundo impacto emocional ^27^.

También, se ha encontrado que el cáncer de mama y la quimioterapia reducen la calidad de vida desde el punto de vista socioeconómico de la familia, evidenciándose cómo aquellas mujeres de niveles socioeconómicos medio o alto y afiliadas al régimen contributivo de salud presentaron los mayores puntajes en la calidad de vida general en contraste con las de nivel socioeconómico bajo afiliadas al régimen subsidiado ^31^. En otro estudio, se determinó que la calidad de vida en mujeres con cáncer de mama en una provincia colombiana era mejor en aquellas afiliadas al régimen contributivo o que recibían apoyo de familiares o amigos, o provenían de un nivel socioeconómico alto ^17^. Se confirma así que un ambiente familiar y socioeconómico favorable mejora la calidad de vida de las mujeres en quimioterapia, pues facilita el cubrimiento de los gastos implícitos y mejora el acceso a los servicios de salud.

En el estudio de Vidal-Cazás ^26^ sobre la calidad de vida en pacientes con cáncer de mama en estadios iniciales y sometidas a tratamiento adyuvante, se encontró que el estado emocional varía durante el tratamiento. Es decir, hay una mayor preocupación al inicio debido a la incertidumbre sobre el estadio del tumor, el pronóstico y la posibilidad de un tratamiento efectivo.

El estado emocional de una paciente resulta de la suma de la calidad de vida previa y de su papel preponderante como madre, esposa y trabajadora, además de aquellas emociones y sentimientos que evolucionan durante el diagnóstico y el tratamiento, así como de los efectos adversos de la quimioterapia. Con base en las dimensiones de la calidad de vida que se ven afectadas, se deben implementar políticas y medidas tendientes a mejorar las condiciones de salud física y emocional de las pacientes. Los factores relacionados con la identidad sexual femenina (feminidad, maternidad, erotismo, papel social) pueden verse afectados de forma variable. Las mujeres con cáncer de mama enfrentan miedos y angustias que requieren un acompañamiento profesional y de la familia, para recuperar y mantener su calidad de vida. El tratamiento del cáncer de mama genera un impacto negativo en la percepción de la calidad de vida de las mujeres, ocasionado principalmente por la reducción de su autoestima y por otras alteraciones en su salud física y mental.

Las limitaciones de este estudio se centran en el hecho de que no se pudo hacer un muestreo probabilístico, por lo que se invitó a participar a todas las mujeres con cáncer de mama que cumplían con los criterios de inclusión y que se presentaron durante el periodo de estudio, lo que puede considerarse como un sesgo de selección del estudio. En el análisis de los resultados, no se pudo establecer el efecto negativo del diagnóstico de cáncer de mamá en la calidad de vida porque no se contó con un grupo de control (mujeres sin diagnóstico de cáncer de mama), por lo que la comparación se hizo en torno a la percepción de la calidad de vida como variable principal del estudio, reconociendo que padecer una enfermedad como esta es un factor determinante en la calidad de vida relacionada con la salud. Tampoco se evaluó el efecto positivo o negativo de los círculos sociales de apoyo de las pacientes, lo que podría estar relacionado con la calidad de vida.

La mayoría de las mujeres presentan una importante disminución de su vida sexual desde el momento del diagnóstico de la enfermedad. Los principales efectos adversos de la quimioterapia, como la alopecia y la delgadez, así como la percepción negativa de la imagen corporal y la feminidad, son causa de la disminución de la calidad de vida. Se evidenció que las pacientes con una red de apoyo establecida reportaban una mejor calidad de vida al tener mejores relaciones interpersonales, más apoyo familiar y social, más afecto, y mejor resiliencia a los efectos del tratamiento. Los síntomas que más impactaban la calidad de vida fueron el dolor, la fatiga y el sueño, por la drástica restricción de la capacidad funcional de las personas y el estrés psicológico potenciado por las náuseas, el vómito, las alteraciones del funcionamiento sexual y la falta de acceso a los servicios de salud.

La valoración de la calidad de la vida es de gran provecho para brindar mejores cuidados y constituye una de las intervenciones más importantes en los pacientes oncológicos, aunada al aumento en la expectativa de supervivencia que ofrecen los nuevos tratamientos. Se recomienda estudiar el número de relaciones sexuales y el placer sexual en estas pacientes y ampliar el estudio para incluir el afrontamiento de su pareja y su familia, pues son elementos que afectan la calidad de vida. Se recomienda implementar un modelo de cuidado integral de la salud en mujeres con cáncer de mama en las instituciones.
